# Duplicate Prescription Patterns in Patients With Substance Use Disorders

**DOI:** 10.1002/prp2.70211

**Published:** 2025-12-17

**Authors:** Johannes Heck, Benjamin Krichevsky, Martin Schulze Westhoff, Konstantin Fritz Jendretzky, Martin Klietz, Stephan Greten, Alexander Glahn, Stefan Bleich, Sebastian Schröder

**Affiliations:** ^1^ Institute for Clinical Pharmacology Hannover Medical School Hannover Germany; ^2^ Institute for General Practice and Palliative Care Hannover Medical School Hannover Germany; ^3^ Interdisciplinary Emergency Department University Hospital Schleswig‐Holstein, Campus Kiel Kiel Germany; ^4^ Department of Psychiatry, Social Psychiatry and Psychotherapy Hannover Medical School Hannover Germany; ^5^ Department of Neurology Hannover Medical School Hannover Germany; ^6^ PRACTIS Clinician Scientist Program, Dean's Office for Academic Career Development Hannover Medical School Hannover Germany

**Keywords:** addiction psychiatry, cross‐sectional study, duplicate prescriptions, medication review, pharmacotherapy safety

## Abstract

This study sought to investigate the frequency and characteristics of duplicate prescriptions (DPs) in psychiatric inpatients with substance use disorders using a DP classification that differentiates between appropriate DPs (ADPs) and potentially inappropriate DPs (PIDPs). The study was conducted as a monocentric retrospective cross‐sectional study on the addiction‐specific ward of the Department of Psychiatry, Social Psychiatry and Psychotherapy of Hannover Medical School, a large university hospital in northern Germany. The outcome measures were the nature and frequency of PIDPs compared with ADPs. Medication records of 1174 patients enrolled between January 2016 and December 2021 were reviewed. The median age of the study population (27.8% female) was 48 years (interquartile range (IQR) 39–56). Patients took a median of 4 drugs (IQR 2–6). 12.8% of the study population were exposed to at least one PIDP (at least one grade‐1 PIDP: 12.0%; at least one grade‐2 PIDP: 1.3%; at least one grade‐3 PIDP: 0.3%). Sedatives were the most common cause of grade‐1 PIDPs, while grade‐2 PIDPs were elicited predominantly by opioids. Nearly one third of the study population (29.0%) displayed at least one ADP. Physicians treating patients with substance use disorders should carefully monitor for PIDPs, particularly PIDPs elicited by sedatives and opioids. Addressing PIDPs may help prevent adverse drug reactions and reduce drug expenses.

## 1 Introduction

Minimization of adverse drug reactions (ADRs) is a critical goal in the context of rational and safe pharmacotherapy, particularly in individuals with addiction disorders [1, 2, 3]. Patients diagnosed with addiction disorders have an increased susceptibility to various health complications, including cardiovascular disease, heart failure, various types of cancer, cirrhosis of the liver, diabetes mellitus, pancreatitis, depression, and anxiety disorders, culminating in an increased risk of premature death [4, 5, 6, 7]. Consequently, people with addiction disorders are often prescribed multiple medications, which in turn presents a notable predisposing factor for the occurrence of ADRs [8, 9]. The consequences of ADRs can be particularly dangerous for people with addiction disorders, given the associated risks of sedation, lactic acidosis, hypoglycemia, orthostatic hypotension, and the potential for gastrointestinal bleeding and liver damage [2].

Duplicate prescriptions (DPs)—among other factors—have been identified as a risk factor for the development of ADRs [10, 11, 12] but there is considerable heterogeneity in the definition of DPs within the medical literature (overview in reference [13]). Mostly developed by pharmacists to assess pharmaceutical interventions [11, 14, 15, 16, 17, 18], the majority of currently existing DP definitions lack applicability in medical settings and are therefore not useful to physicians. Furthermore, the majority of DP definitions characterize DPs as exclusively detrimental to prescribing quality and patient safety, neglecting clinical circumstances in which DPs may be beneficial, such as the evidence‐based and guideline‐supported concurrent administration of two platelet aggregation inhibitors after coronary stent implantation (e.g., low‐dose acetylsalicylic acid plus a P2Y_12_ receptor antagonist) [19]. These inconsistencies, limited clinical utility, and absence of differentiation between therapeutically desired and undesired DPs in the medical literature present substantial challenges to the comparability of studies in the field. Addressing these limitations, Heck and colleagues proposed a novel categorization of DPs, which emphasizes the physician's perspective and distinguishes between appropriate DPs (ADPs) and potentially inappropriate DPs (PIDPs) [20].

ADPs are rational and commonly employed combination treatments, often as add‐on therapy, combining treatment principles with synergistic modes of action, in contrast to PIDPs. The term PIDP was chosen deliberately with reference to the concept of PIMs (potentially inappropriate medications), i.e., drugs with an unfavorable benefit–risk profile in older individuals [21, 22]. PIDPs are further classified into three grades; higher grades denote an increasing degree of inappropriateness [20]. Importantly, it must be emphasized that the degree of inappropriateness of PIDPs as a theoretical model does not necessarily equate to clinical severity.

While the prevalence and characteristics of ADPs and PIDPs have already been studied in geriatric psychiatry [13, 23] and internal medicine (emergency department) [24], the field of addiction psychiatry remains uncharted territory in this respect. Therefore, the present investigation aimed at examining the prevalence and characteristics of DPs in a cohort of patients with substance use disorders receiving treatment in the specialized addiction unit of Hannover Medical School, a large university hospital in northern Germany.

## 2 Methods

### 2.1 Ethics Statement

The study was approved by the Ethics Committee of Hannover Medical School (No. 10764_BO_K_2023) and adhered to the Declaration of Helsinki (1964) and its later amendments.

### 2.2 Study Design and Eligibility Criteria

The present study was designed as a retrospective monocentric cross‐sectional study. The patient cohort was collected sequentially. Patients were eligible for enrolment (i) if they were treated in the addiction unit of the Department of Psychiatry, Social Psychiatry and Psychotherapy of Hannover Medical School between January 2016 and December 2021, (ii) if they suffered from a substance use disorder, (iii) if they were prescribed at least two drugs, and (iv) if they or their legal guardian had provided written informed consent that allowed patient data to be used for research purposes. Hannover Medical School is a university hospital and tertiary care center in northern Germany. The addiction‐specific unit is specialized in the treatment and care of patients with substance use disorders. All patients were inpatients. There were no specific exclusion criteria.

### 2.3 Identification of Demographic and Medication Data

Demographic characteristics, i.e., age, sex, and medical diagnoses, were obtained from patient records. Patients' medications were retrieved from hospital discharge letters.

### 2.4 Definition and Identification of Appropriate Duplicate Prescriptions and Potentially Inappropriate Duplicate Prescriptions

All drugs taken by the patients (both regularly taken drugs and *pro re nata* (PRN) drugs) were analyzed with the aid of the DP classification by Heck et al. [20]. In brief, grade‐1 PIDPs were defined as prescriptions of “two drugs with overlapping or comparable pharmacodynamics”, grade‐2 PIDPs as prescriptions of “two drugs of the same therapeutic class (that is, targeting the same molecular structure)” and grade‐3 PIDPs as prescriptions of “two times the same drug (exceeding the recommended maximum daily dose)” [20]. ADPs, by contrast, were defined as “two drugs with therapeutically desired synergistic effects (that is, established combination treatments)” [20]. Medication charts of enrolled patients were screened for ADPs and PIDPs by an interdisciplinary expert team (JH: clinical pharmacology; BK: internal medicine; KFJ, MK, SG: neurology; MSW, AG, SB, SS: psychiatry).

### 2.5 Outcome Measures

The outcome measures were defined as the frequency and characteristics of PIDPs in psychiatric inpatients with substance use disorders, compared with the frequency of ADPs.

### 2.6 Statistical Analysis

We applied descriptive statistical methods to summarize the data. Since patient characteristics were not normally distributed, quantitative variables are presented as medians with interquartile ranges (IQRs), while categorical variables are shown as absolute and relative frequencies. To assess differences between patients exposed to at least one PIDP (hereon referred to as patients with PIDPs) and those not exposed to PIDPs (hereon referred to as patients without PIDPs), Pearson's chi‐squared test was employed for categorical variables (i.e., variables sex, presence of polypharmacy, and presence of ADPs). For quantitative variables (i.e., variables age and number of drugs), the Mann–Whitney *U* test was used. Two‐sided *p*‐values < 0.05 were considered statistically significant. Given the exploratory nature of our study, no corrections for multiple testing were carried out. Further details on the statistical methods are provided in Table 2. All analyses were conducted with IBM SPSS Statistics 29 (Armonk, New York, USA).

## 3 Results

### 3.1 Study Population and Disease Characteristics

The median age of the study population (*n* = 1174) was 48 years (IQR 39–56; range 18–79), with 27.8% female patients (Table 1). The three most common addiction‐specific psychiatric diagnoses were alcohol use disorder (83.8%), opioid use disorder (25.3%), and cannabis use disorder (18.8%), while the three most frequent psychiatric diagnoses apart from substance use disorders were depression (45.2%), personality disorder (14.7%), and post‐traumatic stress disorder (8.8%). Among somatic comorbidities, arterial hypertension was most prevalent (20.6%), followed by type 2 diabetes mellitus (8.4%) and status post withdrawal seizure (7.1%). Patients' medication regimens comprised a median of 4 drugs (IQR 2–6; range 2–23).

**TABLE 1:** Characteristics of the study population (*n* = 1174).

Category	*n*	%
Sex		
Male	848	72.2
Female	326	27.8
Psychiatric disorders^a^		
Alcohol use disorder	984	83.8
Depression	531	45.2
Opioid use disorder	297	25.3
Cannabis use disorder	221	18.8
Benzodiazepine use disorder	212	18.1
Cocaine use disorder	213	18.1
Personality disorder	172	14.7
Post‐traumatic stress disorder	103	8.8
Multiple substance use disorder	54	4.6
Amphetamine use disorder	45	3.8
Status post withdrawal delirium	37	3.2
Schizophrenia or schizophreniform disorder	35	3.0
Bipolar affective disorder	9	0.8
Dementia	7	0.6
Delirium	5	0.4
Other psychiatric disorder(s)	475	40.5
Somatic disorders^a^		
Arterial hypertension	242	20.6
Type 2 diabetes mellitus	99	8.4
Status post withdrawal seizure	83	7.1
Hypothyroidism	62	5.3
Chronic obstructive pulmonary disease	57	4.9
Epilepsy	39	3.3
Coronary heart disease	36	3.1
Status post stroke (ischaemic or haemorrhagic)	34	2.9
Chronic heart failure	30	2.6
Atrial fibrillation	26	2.2
Urinary tract infection	18	1.5
Intrahospital seizure	4	0.3
Other somatic disorder(s)	789	67.2

^a^ More than one diagnosis was possible per patient.

### 3.2 Potentially Inappropriate Duplicate Prescriptions

12.8% of the study population were exposed to at least one PIDP. As shown in Table 2, there were no statistically significant differences regarding age or sex between patients with and without PIDPs. Regarding PIDP subclassification, 12.0% of patients were exposed to at least one grade‐1 PIDP, 1.3% to at least one grade‐2 PIDP, and 0.3% to at least one grade‐3 PIDP.

**TABLE 2:** Comparison of patients with and without potentially inappropriate duplicate prescriptions.

Category	Total study population	Patients without PIDPs	Patients with PIDPs	*p*
	(*n* = 1174)	(*n* = 1024; 87.2%)	(*n* = 150; 12.8%)	
Median age (IQR)—years	48 (39–56)	48 (39–56)	46.5 (38–55)	0.307^a^
Female sex—% (no.)	27.8 (326)	27.2 (279)	31.3 (47)	0.297^b^
Median number of drugs (IQR)	4 (2–6)	3 (2–5)	5 (4–7)	**< 0.001** ^a^
Polypharmacy—% (no.)	36.1 (424)	33.1 (339)	56.7 (85)	**< 0.001** ^b^
Patients with ADPs—% (no.)	29.0 (340)	29.5 (302)	25.3 (38)	0.294^b^

*Note:* Patients with PIDPs were defined as those exposed to at least one PIDP, while patients without PIDPs had no such exposure. Patients with ADPs were identified as those who had at least one ADP. Polypharmacy was defined as the simultaneous use of five or more different drugs.

Abbreviations: ADP, appropriate duplicate prescription; IQR, interquartile range; no., number; PIDP, potentially inappropriate duplicate prescription.

^a^ Quantitative variables were tested for normality using the Shapiro–Wilk test. Since the data were not normally distributed, differences between patients with and without PIDPs were analyzed using the two‐sided Mann–Whitney *U* test for independent samples [25]. Statistically significant *p*‐values (i.e., *p* < 0.05) are highlighted in bold.

^b^ Pearson's chi‐squared test was employed to assess the relationship between the presence of PIDPs and factors such as sex, polypharmacy, or presence of ADPs. Statistically significant *p*‐values (i.e., *p* < 0.05) are highlighted in bold.

In total, 168 PIDPs were identified in the study population. Most PIDPs were of grade 1 (89.2%; 150/168), with sedatives being the primary contributors (46.0%; 69/150). Among those, the combination of pipamperone and low‐dose quetiapine was the most prevalent (14.5%; 10/69) (Table 3). Opioids were most frequently responsible for grade‐2 PIDPs (40.0%; 6/15), with fentanyl + oxycodone (26.7%; 4/15) being the leading combination. Notably, all grade‐3 PIDPs involved different drug classes (angiotensin receptor blockers, immunosuppressants, and vitamins).

**TABLE 3:** Absolute and relative frequencies of potentially inappropriate duplicate prescriptions detected in the study population.

PIDPs	*n*	%
Grade‐1 PIDPs	150	100
Sedatives	69	46.0
Agomelatine + low‐dose quetiapine	2	2.9
Agomelatine + pipamperone	3	4.3
Agomelatine + valerian	1	1.4
Amitriptyline + pipamperone	1	1.4
Chlorprothixene + lorazepam	3	4.3
Chlorprothixene + low‐dose mirtazapine	2	2.9
Chlorprothixene + pipamperone	1	1.4
Diazepam + valerian	1	1.4
Doxepin + melperone	1	1.4
Doxepin + low‐dose mirtazapine	2	2.9
Flunitrazepam + low‐dose mirtazapine	1	1.4
Hydroxyzine + low‐dose quetiapine	1	1.4
Levomepromazine + low‐dose mirtazapine	4	5.8
Lorazepam + pipamperone	1	1.4
Lorazepam + zolpidem	5	7.2
Low‐dose mirtazapine + pipamperone	6	8.7
Low‐dose mirtazapine + low‐dose quetiapine	4	5.8
Low‐dose mirtazapine + valerian	2	2.9
Olanzapine + pipamperone	1	1.4
Oxazepam + pipamperone	9	13.0
Pipamperone + promethazine	2	2.9
Pipamperone + low‐dose quetiapine	10	14.5
Pipamperone + valerian	4	5.8
Pipamperone + zolpidem	1	1.4
Pipamperone + zopiclone	1	1.4
Antidepressants	44	29.3
Agomelatine + mirtazapine	3	6.8
Agomelatine + moclobemide	1	2.3
Agomelatine + opipramol	1	2.3
Agomelatine + sertraline	2	4.5
Amitriptyline + duloxetine	1	2.3
Amitriptyline + sertraline	2	4.5
Citalopram + doxepin	1	2.3
Citalopram + trimipramine	1	2.3
Clomipramine + paroxetine	2	4.5
Doxepin + duloxetine	2	4.5
Doxepin + fluoxetine	1	2.3
Doxepin + sertraline	2	4.5
Doxepin + venlafaxine	7	15.9
Fluoxetine + opipramol	3	6.8
Fluoxetine + trazodone	1	2.3
Mirtazapine + opipramol	1	2.3
Opipramol + paroxetine	2	4.5
Opipramol + venlafaxine	1	2.3
Sertraline + trazodone	1	2.3
Tianeptine + venlafaxine	5	11.3
Trimipramine + venlafaxine	4	9.1
Analgesics	15	10.0
Ibuprofen + metamizole	13	86.7
Metamizole + paracetamol	2	13.3
Acid‐blocking agents	6	4.0
Esomeprazole + sucralfate	1	16.7
Pantoprazole + magaldrate	3	50.0
Pantoprazole + sucralfate	2	33.3
Antipsychotics	6	4.0
Amisulpride + clozapine	1	16.7
Aripiprazole + olanzapine	1	16.7
Chlorprothixene + quetiapine	2	33.3
Clozapine + quetiapine	1	16.7
Quetiapine + risperidone	1	16.7
Cardiovascular agents	5	3.3
Beta‐acetyldigoxin + verapamil	1	20.0
Bisoprolol + clonidine	1	20.0
Candesartan + ramipril	1	20.0
Carvedilol + doxazosin	1	20.0
Ramipril + valsartan	1	20.0
Laxatives	2	1.3
Lactulose + macrogol 3350	2	100
Antiepileptic drugs	1	0.7
Levetiracetam + valproate	1	100
Platelet aggregation inhibitors	1	0.7
Acetylsalicylic acid + cilostazol	1	100
Vitamins	1	0.7
Vitamin B‐Komplex + Dreisavit	1	100
Grade‐2 PIDPs	15	100
Opioids	6	40.0
Fentanyl + oxycodone	4	66.7
Hydromorphone + levomethadone	1	16.7
Levomethadone + tramadol	1	16.7
Inhalatives	3	20.0
Formoterol + salbutamol	3	100
Antipsychotics	2	13.3
Chlorprothixene + pipamperone	1	50.0
Melperone + pipamperone	1	50.0
Antihistamines	1	6.7
Cetirizine + desloratadine	1	100
Antimicrobial agents	1	6.7
Flucloxacillin + penicillin	1	100
Diuretics	1	6.7
Hydrochlorothiazide + indapamide	1	100
Sedatives	1	6.7
Lorazepam + oxazepam	1	100
Grade‐3 PIDPs	3	100
Angiotensin receptor blockers	1	33.3
Candesartan + candesartan	1	100
Immunosuppressants	1	33.3
Mycophenolate mofetil (CellCept) + mycophenolic acid (Myfortic)	1	100
Vitamins	1	33.3
Vitamin B‐Komplex + Dreisavit	1	100

*Note:* Relative frequencies add up to 100% within all subgroups.

Abbreviation: PIDP, potentially inappropriate duplicate prescription.

### 3.3 Appropriate Duplicate Prescriptions

In almost one‐third of patients (29.0%), at least one ADP was present (hereon termed patients with ADPs). The proportion of patients with ADPs was slightly lower among patients with PIDPs than among those without PIDPs, though the difference was not statistically significant (25.3% (38/150) vs. 29.5% (302/1024); *p* = 0.294).

### 3.4 Polypharmacy

Polypharmacy, defined as the use of five or more different medications simultaneously [26], was observed in 36.1% of the study population. Patients with PIDPs were significantly more likely to be affected by polypharmacy than those without PIDPs (56.7% (85/150) vs. 33.1% (339/1024); *p* < 0.001). Accordingly, patients with PIDPs took a higher number of medications than patients without PIDPs (median 5 drugs (IQR 4–7) vs. 3 drugs (IQR 2–5); *p* < 0.001).

## 4 Discussion

This study sought to analyze the frequency and characteristics of DPs among psychiatric inpatients with substance use disorders treated at a university hospital in Germany. To our knowledge, this is the first investigation examining DPs in this patient population. The study was based on medication chart reviews conducted by an interdisciplinary expert team comprising specialists from psychiatry, neurology, internal medicine, and clinical pharmacology. Among 1174 patients (median age 48 years, 27.8% female), at least one PIDP was present in 12.8%. More specifically, 12.0%, 1.3%, and 0.3% of the study population were exposed to at least one grade‐1, grade‐2, and grade‐3 PIDP, respectively. Sedatives were the most important drivers of grade‐1 PIDPs, whereas grade‐2 PIDPs were predominantly elicited by opioids. The causes of grade‐3 PIDPs were heterogeneous, ranging from angiotensin receptor blockers to immunosuppressants to vitamins.

The proportion of patients affected by PIDPs in the present study lay in the range of previous analyses by Heck et al. in the emergency department (10.9%) [24] and Heinze et al. in the Austrian population taking antihypertensive, antilipidemic, or antidiabetic agents (13%–15%) [27], but was markedly lower than in a study by Schulze Westhoff et al. in geriatric psychiatry (43.1%) [13]. In the present study, the number of prescribed drugs as well as the proportion of patients affected by polypharmacy were higher among patients with PIDPs compared to patients without PIDPs, similar to Heck et al. [24] and Schulze Westhoff et al. [13]. Interestingly, nearly one‐third of our study population displayed at least one ADP, and we observed a trend towards a lower proportion of patients with ADPs among patients with PIDPs compared to those without PIDPs, albeit not statistically significant. Heck et al. [24] and Schulze Westhoff et al. [13], by contrast, did report significantly lower proportions of patients with ADPs among patients with PIDPs than among those without PIDPs. Clear distinctions between ADPs and PIDPs are clinically relevant since previous studies most often failed to discriminate between beneficial and potentially harmful DPs [13]. Most authors regarded DPs solely as detrimental to patients' health, a view that is challenged by the results of our study. When comparing the findings of our investigation to those of other studies in the field, it is important to bear in mind that there exists a wide variety of terminologies and definitions of DPs [13], subsequently indicated by the use of *italics*. This lack of standardization significantly impedes the comparability of results across different studies. Moreover, all of the studies discussed below examined settings other than addiction psychiatry, which further limits comparability.

In a cross‐sectional prospective study conducted in 97 Italian internal medicine and geriatric wards, the proportion of patients (average age 79 years; 52% female) exposed to at least one *therapeutic duplicate* (defined as “at least two drugs of the same therapeutic class prescribed simultaneously to a patient”) increased significantly from hospital admission (2.5%) to discharge (3.4%) [28]. Similar to our investigation, psychotropic drugs—most notably antidepressants, antipsychotics, and hypnotic sedatives—were among the drugs most frequently involved in *therapeutic duplicates* [28]. Of note, 86.8% of patients discharged with at least one *therapeutic duplicate* were still receiving them three months later, suggesting high persistence rates of *therapeutic duplicates*, even if prescriptions were made by specialists (internists, geriatricians) [28].

In a cross‐sectional retrospective study conducted in the King Khaled Hospital in Saudi Arabia, *therapeutic duplication of medication* accounted for 6.1% of all medication errors detected between 2018 and 2020 [29]. However, neither a definition for *therapeutic duplication of medication* nor the involved drugs/drug classes was reported, representing substantial limitations [29].

In a nationwide register‐based, repeated cross‐sectional study of older adults dispensed drugs at community pharmacies in Sweden between 2006 and 2021, a *drug duplication* was defined as “a ≥ 30‐day overlap of two dispensations of drugs with the same 5th level (chemical substance) Anatomical Therapeutic Chemical (ATC) classification, but with different brand names, within a 3‐month period each year” [30]. This definition is comparable—although not identical—to our definition of a grade‐3 PIDP. While the prevalence of potential *drug duplications* was considerably higher among the older general population in Sweden than the frequency of grade‐3 PIDPs in our study cohort of comparatively young patients with substance use disorders (5.8% in 2006 to 10.9% in 2021 vs. 0.3%), it is remarkable that two of the three drug groups most frequently implicated in *drug duplications* in Sweden in 2021 were similar to the drug groups responsible for grade‐3 PIDPs in our investigation, namely B vitamins and angiotensin receptor blockers, more specifically candesartan [30]. This observation suggests that while our study population was select (psychiatric inpatients with substance use disorders in a single hospital), some of the findings of the present investigation might be of medical relevance in a broader context beyond addiction psychiatry.

Trenaman and colleagues examined *drug duplications* (defined as “2 drugs from the same class” according to the Screening Tool of Older Persons' Prescriptions (STOPP) criteria, specifically concurrent use of two nonsteroidal anti‐inflammatory drugs (NSAIDs), selective serotonin reuptake inhibitors (SSRIs), loop diuretics, angiotensin‐converting enzyme inhibitors (ACEIs), or anticoagulants) among older adults with dementia in Nova Scotia, Canada [31]. *Drug duplications* were found in 3.5% of anticoagulant users, 2.0% of ACEI users, 1.7% of NSAID users, 1.0% of SSRI users, and 0.07% of loop diuretic users [31]. On the one hand, similar to our study, no sex differences were observed. On the other hand, PIDPs containing anticoagulants, ACEIs, SSRIs, or loop diuretics were not present in our study. PIDPs containing non‐opioid analgesics in our study occurred between ibuprofen and metamizole and between metamizole and paracetamol but not between different NSAIDs. One PIDP containing diuretics occurred between hydrochlorothiazide and indapamide but loop diuretics were not involved. Finally, PIDPs containing antidepressants exclusively occurred between different subclasses of antidepressants (e.g., a tricyclic antidepressant (such as amitriptyline) plus an SSRI (such as sertraline)) but not within the same subclass of antidepressants. The differences between Trenaman et al. and our study most likely reflect heterogeneities in methodology (STOPP criteria [31] vs. DP classification by Heck et al. [20]), age profile (average age 81 [31] vs. 48 years) and disease characteristics of the patient cohorts (dementia [31] vs. substance use disorder as the leading psychiatric diagnosis).

Another study that used a DP definition based on STOPP criteria was conducted in a psychiatric hospital in The Netherlands [32]. 36.6% of patients in that study were prescribed *duplicate class drugs*, most frequently benzodiazepines, antipsychotics, and antidepressants [32]. Similar to Trenaman et al., the patients in the Dutch study were markedly older than in our investigation (average age 75 vs. 48 years) and displayed a different profile of psychiatric disorders (fewer substance‐related disorders, more cases of dementia and psychotic disorders) [32].

A strength of our study is the large number of medication chart reviews, which were conducted by an interdisciplinary expert team comprising specialists from psychiatry, internal medicine, neurology, and clinical pharmacology. Each medication regimen was analyzed manually, and the team then decided whether a detected DP should be considered appropriate (i.e., ADP) or potentially inappropriate (i.e., PIDP). Hence, the same drug combination (e.g., an antipsychotic plus a benzodiazepine) could be considered appropriate in one patient and potentially inappropriate in another. We are convinced that utilization of the DP categorization by Heck et al. [20] minimized subjective bias and abrogated the blending of clinically beneficial and detrimental DPs, which was a major limitation of previous works. On the other hand, the retrospective and monocentric design clearly represents limitations. Our study was carried out at a single university hospital; therefore, the results may not generalize to other settings in which patients with substance use disorders are treated.

Anxiety symptoms are frequent in patients with substance use disorders and may have contributed to the observed pattern of sedative prescriptions. Anxiety disorders, however, were not systematically coded as a separate diagnostic entity in the present dataset. In Germany, current guidelines advocate non‐benzodiazepine pharmacotherapy and evidence‐based psychotherapeutic approaches for anxiety management in addiction contexts [33].

Of note, the combination of chlorprothixene and pipamperone was rated as a grade‐1 PIDP on one occasion and as a grade‐2 PIDP in a different case (Table 3). This circumstance was due to the fact that psychotropic drugs often target multiple molecular structures. In the first case, the antagonistic activity of both chlorprothixene and pipamperone at dopamine D_2_ receptors, which increases the risk of extrapyramidal symptoms, led to the categorization as a grade‐2 PIDP. In the second case, the sedating properties of chlorprothixene and pipamperone, which are not attributable to a single molecular target structure in the central nervous system, led to the categorization as a grade‐1 PIDP. This example demonstrates that while the DP classification by Heck et al. [20] is simple and ready‐to‐use, it also has limitations. Yet, the question of whether a specific drug combination should be rated as a grade‐1 or grade‐2 PIDP is rather of academic nature; in clinical practice, it is much more important that a duplicate prescription *is* recognized as potentially inappropriate. The DP classification by Heck et al. might aid clinicians in this context [20].

Similar to DPs, the term polypharmacy can be differentiated into appropriate and inappropriate polypharmacy [34]. A limitation of the present work is that for the statistical analyses, polypharmacy was only used as a quantitative parameter (defined as the coadministration of five or more different drugs) while qualitative aspects were not taken into account. Future studies should consider qualitative aspects not only for DPs but also for polypharmacy.

The most important limitation of our study is the absence of information about the frequency in which PIDPs actually elicited ADRs. Such an analysis was not possible within the framework of the present study due to its cross‐sectional and retrospective nature. Anecdotal evidence suggests that PIDPs are clinically relevant. For instance, Ascenção and colleagues described the case of a 90‐year‐old woman who was started on rivaroxaban despite already taking edoxaban, leading to significant bleeding [12]. *Therapeutic duplications* of anticoagulants were analyzed more systematically by Rahmanzade et al. in a tertiary referral hospital in Switzerland [35]. The authors detected *therapeutic duplications* of anticoagulants in 0.8% of patients who were under anticoagulation and in 6.7% of patients treated with direct oral anticoagulants, leading to hemoglobin‐relevant bleeding in 7.4% of cases [35]. Even though PIDPs involving anticoagulants were not observed among patients with substance use disorders in the present investigation, future prospective follow‐up studies should systematically investigate the clinical relevance of PIDPs in this patient population—with a focus on PIDPs elicited by sedatives and opioids—by using established ADR causality assessment tools [36, 37].

The DP classification used in this study was already integrated into a German textbook of geriatrics [38] and is implemented into teaching of medical students and continued medical education for physicians at Hannover Medical School. Only through dissemination and education will it be possible to raise awareness for the problem of PIDPs. In a Quality Improvement Project in the United Kingdom, Huynh and Rajendran impressively demonstrated that it was possible to reduce the frequency of undesired *therapeutic duplications* (defined as “the practice of prescribing multiple medications for the same indication or purpose without a clear distinction of when one agent should be administered over another”) on general surgical wards from 9% to 3.12% through repeated cycles of education and audits of the medical team (Plan–Do–Study–Act model) [39]. Examples of *therapeutic duplications* detected and rectified mostly corresponded to grade‐2 (e.g., lansoprazole PO + pantoprazole IV) and grade‐3 PIDPs (e.g., oxycodone prescribed as tablets and as solution) [39].

Besides education, it will be crucial to integrate PIDP alerts into electronic prescribing systems in the future. First results in this field, however, have not been entirely convincing. In a pilot study in the United States, the mean detection rate of *therapeutic duplications* by computerized physician order entry (CPOE) systems with clinical decision support (CDS) in test cases of unsafe medication orders was only 39.3% [40]. *Therapeutic duplications* were defined as “medication combinations [that] overlap therapeutically (same agent or class)”, e.g., “use of clonazepam and lorazepam together” [40]. While this definition encompassed grade‐2 and grade‐3 PIDPs according to Heck et al. [20], it did not explicitly discriminate between therapeutically desired and undesired DPs, underscoring again the complexity of the topic.

A major problem of current CPOE‐CDS systems remains a high rate of clinically irrelevant alerts. According to a systematic review by Poly et al., 46%–96% of alerts issued by CPOE‐CDS systems are overridden by users [41]. In detail, the rate of overridden *drug duplicate* alerts ranged between 28.6% and 51.9%, and overriding those alerts was considered clinically appropriate in 82% to 98% of cases, suggesting an overall low specificity of *drug duplicate* alerts [41]. Reasons for overriding *drug duplicate* alerts were—among others—“combination therapy indicated”, “patient requires different strengths of the same drug” and “new evidence supports duplicate therapy of this type” [41], indicating that CPOE‐CDS systems currently do not sufficiently differentiate between ADPs and PIDPs. Strikingly, in a Web‐based survey in a teaching hospital in Sydney, Australia, 47% of participating physicians stated that they would remove *therapeutic duplication* alerts from electronic medical records, mostly because those alerts were issued too frequently and were too clinically unimportant [42]. In the future, implementing a DP classification in CPOE‐CDS systems that takes physicians' views into account [20] appears urgently warranted. A promising finding in this context stems from a cross‐sectional study conducted in community pharmacies in Egypt [43]. Kassem and colleagues detected a significantly lower number of *drug duplication* medication errors in pediatric outpatients when prescriptions were computerized instead of handwritten [43]. However, no thorough distinction between *duplications (same class)* and *active ingredient duplications* was made in that study [43], impeding a direct comparison to different grades of PIDPs [20]. A single‐center pilot study in the United States showed that it was possible to reduce *therapeutic duplications* (defined as “the presence of multiple agents prescribed for the same indication without clarification for when each should be used”) of PRN paracetamol and ibuprofen through implementation of discrete PRN reasons specifying “first line” and “second line” in a CDS system [44]. Thereby, prescribing physicians were induced to reflect on the appropriateness of DPs. As a limitation, the authors stated that “only (…) acetaminophen and ibuprofen therapeutic duplications were evaluated. The potential impact of therapeutic duplication on other medications such as opioids and sedatives is much larger.” [44] This conclusion is fully supported by the results of our study, in which sedatives and opioids were detected as major drivers of PIDPs.

Although the clinical implications of PIDPs are not yet fully established, we suggest that physicians who treat patients with substance use disorders pay special attention to PIDPs, particularly PIDPs elicited by sedatives and opioids. Termination of PIDPs owing to sedatives and opioids may not only reduce the risk of excessive sedation, cognitive impairment, and respiratory depression, but may also save healthcare expenditures elicited by unindicated or unnecessary drugs. A prospective cohort study in the outpatient pharmacy of a quaternary care hospital in India demonstrated that termination of *therapeutic duplications* (defined as “inappropriate duplication of therapeutic group or active ingredient”, largely equivalent to the definitions of grade‐2 and grade‐3 PIDPs, respectively [20]), which accounted for 43.4% of all detected prescribing errors, led to significant cost reductions [45]. If grade‐1 PIDPs were additionally considered as part of a medication review strategy to minimize PIDPs, the cost‐saving potential might even be greater. Besides potential minimization of ADRs, future studies should therefore also evaluate the cost‐saving effect of PIDP termination in psychiatric inpatients with substance use disorders.

## Author Contributions

**Johannes Heck:** conceptualization; data curation; investigation; methodology; formal analysis; visualization; writing – original draft; writing – review and editing. **Benjamin Krichevsky:** conceptualization; data curation; investigation; methodology; formal analysis; visualization; writing – original draft; writing – review and editing. **Martin Schulze Westhoff:** investigation; validation; writing – review and editing. **Konstantin Fritz Jendretzky:** investigation; validation; writing – review and editing. **Martin Klietz:** investigation; validation; writing – review and editing. **Stephan Greten:** investigation; validation; writing – review and editing. **Alexander Glahn:** investigation; validation; writing – review and editing; supervision. **Stefan Bleich:** investigation; validation; writing – review and editing; supervision. **Sebastian Schröder:** conceptualization; data curation; investigation; methodology; formal analysis; visualization; writing – original draft; writing – review and editing.

## Conflicts of Interest

The authors declare no conflicts of interest.

## Data Availability

The data that support the findings of this study are available upon reasonable request from the corresponding author.
